# Subjective Perception of Work and the Home Care Workers’ Depression

**DOI:** 10.3390/ijerph192416894

**Published:** 2022-12-16

**Authors:** Yu Zhang, Hanjin Zhang, Yihui Su

**Affiliations:** 1 School of Marxism, University of Shanghai for Science and Technology, No. 334 Jungong Road, Shanghai 200093, China; 2 Department of Sociology, College of Humanities, Shanghai University of Finance and Economics, No. 777 Guoding Road, Shanghai 200433, China

**Keywords:** home worker, depression, subjective perception of work

## Abstract

The care crisis has become a global trend, and the demand for child and elderly care is increasing worldwide. The increasing number of home care workers plays a significant role in meeting this demand in developing countries. The mental health of these workers is associated with the quality of care they provide, which has rarely been studied. This paper explored the factors that affect home care workers’ depression, including their socio-demographic characteristics, working environment, relationship with clients, social support networks, economic burden, and subjective perceptions of discrimination and work. It utilized data from the Shanghai Domestic-work Professionalization Survey (SDPS), which was conducted among four types of in-home caregivers (*n* = 1000) in Shanghai over a period ranging from May to September 2021. The results show that the variables of marriage, education, self-reported health, relationship with client, economic burden, and subjective perceptions of discrimination and care work are significantly associated with home care workers’ level of depression. However, the variables of gender, age, household registration, and work environmental factors have no significant effect on their level of depression, which differs from the findings of previous studies on care workers in other institutions.

## 1. Introduction

The care crisis has become a global trend, and the demand for child and elderly care is increasing worldwide. In developed countries in Europe and North America, child and elderly care is usually provided by female immigrants from developing countries such as the Philippines and Indonesia in the form of paid care work [1,2,3]. Most of this commercial care work is home-based and informal [4,5]. Likewise, the demand for child and elderly care in developing countries has boomed during these years. In developing countries, female migrants from rural areas provide paid care services for urban families [6]. Their care service is also home-based and in the informal sector [7,8]. In contrast to caregivers in hospitals, who are well studied in existing research, these female rural migrants’ mental health has not received attention from scholars. To contribute to the stream of current studies of caregivers, this paper explored the depression level of these female rural migrant care workers and the factors affecting their mental health in developing countries.

In China, the care industry has been booming during recent years. In 2019, the annual turnover of the care industry in China reached 690 billion RMB in 2018, and the number of care workers exceeded 30 million [9]. Similar to other developing countries, these care workers are mainly female migrants from rural areas. They are mainly middle-aged and low-educated. A large-scale random-sample survey of domestic workers conducted in 2019 revealed that only 1.6% of care workers in Beijing have labor contracts [10,11]. Other studies show that these female rural migrant care workers usually work overtime, lack leisure time, and sometimes have to face abuse from clients [12,13,14,15,16,17]. Although scholars have paid attention to these female rural migrant care workers’ working conditions and relationships with their clients, the mental health of these workers has not received attention from scholars. However, studies show that a decrease in care workers’ mental health might have a detrimental influence not only on their own physical health [18], but also on the quality of care they are able to provide [19]. As they comprise a significant dimension of mental health, female rural migrant care workers’ depression levels should be carefully examined. Studies show that caregivers tend to experience a variety of depressive symptoms, such as feelings of anxiety, loneliness, fearfulness, isolation, fatigue, and sleep deprivation [20]. The Center for Epidemiologic Studies Depression Scale (CES-D) developed by Radloff could be used to measure the self-reported depression of caregivers [21].

Existing studies examined the influence of multiple factors on caregivers’ depression levels, including socio-demographic characteristics, the working environment, and social relationships. These studies have found racial and ethnic differences in the relationship between socio-demographic variables and caregiver depression [22,23,24]. Furthermore, the working environment is regarded as an important factor affecting caregivers’ depression. Because home care work is conducted in a private place without protection from institutions, home care workers must depend on their own resources to deal with violence and abuse in their working environment [25]. Therefore, abuse experienced in the working environment, such as personal injuries, sexual harassment, and verbal abuse, increases home care workers’ depression levels [26]. Furthermore, studies show that care receivers with a high degree of depression might increase their caregivers’ depression levels through direct interaction [27]. Discrimination from clients also contributes to the caregiver’s depression levels [28]. In contrast, having good relationships with clients might relieve caregivers’ stress levels [12,13,14,15]. In addition, caregivers’ social relations with their friends or their community might provide social support for them to cope with stress and anxiety [27].

Although these studies revealed the influence of socio-demographic characteristics, the working environment, and relationships with clients on caregivers’ depression levels, they ignored the relationship between caregivers’ depression levels and the subjective perception of their care work. Research on workers in formal sector shas shown that subjective perceptions of their care work, such as job satisfaction, have a negative impact on employees’ depression [29,30]. Although the relationship between informal home care workers’ depression levels and the subjective perception of their care work has not been adequately explored, previous studies on formal institutional caregivers such as nurses suggested that a high level of job satisfaction might diminish the negative effects of their emotional exhaustion [31], while a low level of job satisfaction might lead to occupational burnout [32,33,34,35,36,37]. Therefore, to fill in the gap, this study emphasized the analysis of the association between informal home care workers’ depression levels and their subjective perception of work.

Furthermore, previous studies of caregivers’ depression levels focused on caregivers who are family members or professionals who care for disabled patients [38,39,40]. These findings may not be applicable for in-home care workers who are female rural migrants caring for children and elderly people because this group of individuals is quite different from the two former groups. First, home care workers in China are mostly rural migrant women who do not receive a high level of education or as much training as professionals who care for disabled patients [10]. Even some regional trade unions and domestic companies in China provided training on emotional regulation strategies to workers in order to relieve their anxiety and pressure, and seldom research explores the effect of these trainings. Due to these differences, their strategies for coping with stress and anxiety in the workplace might be different. Second, their relationships with their clients are also different from those that the care receivers have with their family members who provide care. In contrast to family members who engage in care work, rural migrant women who provide commercial care work usually face discrimination from care receivers [13,14]. Furthermore, the informality of home care workers also leads to precariousness in their daily life. Studies show that some rural migrant families encounter strong economic burden [8,12,13,14,15]. However, the differences have not received enough attention from scholars, as there are very few empirical studies that examine the factors that are associated with the level of depression of these rural migrant women. Therefore, this study explored the factors that affect in-home caregivers’ depression levels using respondent-driven sampling of in-home caregivers (*n* = 1000) drawn from the Shanghai Domestic-work Professionalization Survey (CDPS). It aimed to facilitate policy-makers such as the All-China Women’s Federation (ACWF) in improving the mental health of in-home caregivers who are female migrants. It also contributes to the previous literature in two aspects. First, we focused on female migrants’ depression levels, which is an issue that has been neglected in the previous studies of caregivers in developing countries. Second, our work explored the relationship between female migrants’ subjective perception of their care work and their depression levels. In this way, our work may provide recommendations for improving female migrants’ mental health as it relates to their subjective feelings toward their work.

## 2. Materials and Methods

### 2.1. Data and Design

This study utilized data dawn from the Shanghai Domestic-work Professionalization Survey (CDPS). The CDPS was funded by The National Social Science Fund of China and was conducted from early May to early September 2021 in Shanghai based on the respondent-driven sampling approach. The aim of the research was to identify factors that may influence home care workers’ depression levels. The respondents consisted of home care workers who worked for private households for more than 3 months, including home care workers who only provide maternal care, those who only provide child care, those who only provide elderly care, housekeepers, and cleaners in Shanghai. A cleaner only cleans the house, but a housekeeper’s work might include both house cleaning and child care. The utilized questionnaire included items on the respondents’ basic personal information, work status, employment status, subjective perception of their care work, health status, employers’ situation, etc. In this study, we used the respondent-driven sampling (RDS) method based on recommendations made by 12 home care workers who we called the “initial seeds”. These 12 initial seeds were recruited according to previous fieldwork conducted on home care workers. They included 1 housekeeper, 3 cleaners, 2 elderly care workers and 6 maternal-child care workers. Each respondent was rewarded with RMB 50 (approximately USD 7.70) for answering the questionnaire and with RMB 10 (approximately USD 1.50) for each successful recommendation. To record referral chain information, each participant was assigned three coupons labeled with a specific number. In this way, the CDPS acquired a total sample size of 1000, and each characteristic had a design effect of greater than two, which enhanced the confidence level of the sample.

### 2.2. Variables

The socio-demographic characteristic variables included gender, age, marital status (married, divorced, widowed, unmarried), household registration (urban or village), self-reported health (very good, good, neutral, bad, very bad), and level of education (no education completed, primary school, junior high school, high school, vocational high school, technical secondary school, college, undergraduate, and above). In terms of working environment, respondents were asked about their annual income, number of rest days per month, living condition (living with the clients or not), working hour arrangement (lived-in, day shift, hourly), contractual status (no contract, have contract), surveillance (no surveillance, surveillance by camera, unclear), and whether they received training on emotional regulation strategies (have not received, have received). With regard to the type of care, respondents were asked about the types of care they provide (maternal care, child care, elderly care, housekeeper, cleaner). In terms of social relationships, respondents were asked about their relationships with their clients (good, neutral, bad) and their social supportive networks, which are the number of friends they contact more than once per month. Regarding life-related factors, respondents were asked about whether they have economic burden (no economic burden, have economic burden). Last, in terms of their subjective perception of work, respondents were asked to describe their subjective perception of discrimination (discrimination, no discrimination) and how they like their work (like very much, like, neutral, do not like, do not like very much).Some of the categories of these variables were merged in the regression model to achieve a more balanced distribution of the sample size by category and a better model result. The merged categories are reported in the following section.

The depression level of each respondent was estimated using the Center for Epidemiologic Studies Depression Scale (CES-D) suggested by Radloff [21]. To eliminate redundant items in the original version, Andresen, Malmgren, Carter, and Patrick utilized item-total correlations and chose only 10 items to develop a simplified version called the CES-D 10 [41]. The 10 items in the CES-D 10 include “I was bothered by things that usually don’t bother me”, “I had trouble keeping my mind on what I was doing”, “I felt depressed”, “I felt that everything I did was an effort”,” I felt hopeful about the future (reverse scored)”, “I felt fearful”, “My sleep was restless”, “I was happy(reverse scored)”, “I felt lonely”, and “I could not ‘get going’”. Answers to the CES-D 10 questions are coded as “0” for “rarely or none of the time (less than 1 day)”, “1” for “some or a little of the time (1–2 days)”, “2” for “occasionally or a moderate amount of time (3–4 days)”, and “3” for “most of time (5–7 days)”.

The final score was summed by totaling all the items scored after reversing the positive mood items (scale ranging from 0 to 30). Higher scores illustrate greater degrees of a depressed mood.

### 2.3. Statistical Analysis

We first conducted a descriptive analysis of the variables related to socio-demographic characteristics, working environment, social support network (numbers of contacts per month), relationships with clients, home care workers’ depression, and their subjective perceptions of discrimination and work. Multiple regression models were then constructed to analyze the relationship between home care workers’ depression levels and their socio-demographic characteristics, working environment social support network (numbers of contacts per month), relationships with clients, their subjective perception of discrimination, and work. All statistical analyses were conducted using SPSS 26.

## 3. Results

Table 1 shows the descriptive analysis of the independent variables, including socio-demographic characteristics, working environment, social support network (number of contacts per month), relationships with clients, home care workers’ economic burden, their depression, their subjective perception of discrimination, and work. The proportions of male and female respondents in Shanghai were 2.8% and 97.2%, respectively, which suggests that home care workers in Shanghai are mainly women. The average age of the respondents in Shanghai was 50 years, and the standard deviation was 6.4. Therefore, home care workers in Shanghai are an older group. A total of 21.7% of the Shanghai respondents had an urban household registration, while78.3% had a rural household registration. The respondents’ most common marital status was married, accounting for more than 90% of the sample. Almost 27.2% of the respondents had only completed primary school, 46.7% had completed junior high school, and 97.5% had completed high school or below. Therefore, the education level of home care workers in Shanghai is limited. Furthermore, the respondents’ self-assessments about their physical health were concentrated in three options: very good, good, and neutral, which when combined accounted for 96.8% of the total; this indicates that Shanghai home care workers are generally in good health.

The respondents’ average annual income was RMB 64,123 (equivalent to USD 9201). The respondents’ average number of rest days per month was 3.4. Almost 56% of the respondents held a labor contract, but almost 44% had no contract. Regarding the type of care provided, 7.3% of the respondents were maternal care workers, 13.3% were child care workers, 28.5% were elderly care workers, 36.1% were housekeepers, and 14.2% were cleaners. In terms of working hour arrangement, 34.2% of the respondents were live-in workers, 14.9% were day-shift workers, and 50.9% were hourly workers. Moreover, 34.7% of the respondents lived with their client, while 65.3% did not live with their client. A total of 41.1% of the respondents reported that they were under surveillance by camera, 50.3% were not under surveillance, and 8.6% were not sure whether they were under surveillance. With regard to the training on emotional regulation strategies, only 35.6% of the respondents reported that they received this kind of training provided by their company.

In terms of the relationship with their clients, 89.4% of the respondents reported having a good relationship with their clients. On average, the number of people they contacted per month was reported to be 13. Regarding home care workers’ daily life, 16.9% of the respondents reported that they have economic burden.

Regarding subjective discrimination, 51.1% of the respondents reported that they had not encountered discrimination, while 48.9% reported that they had encountered discrimination. In terms of their subjective perception of care work, 8.9% of the respondents reported that they did not like care work, while 71.8% reported that they liked care work.

Table 2 shows the average score of home care workers’ depression in each item of CES-D10. The results show that the average score in each item was less than 1. The mean value of the final score, which was summed by totaling all the items, is 4.650. This illustrates the lower level of home care workers’ depression.

Table 3 shows the results of the multivariable linear regression model using the depression index with a confidence level of 95%. This model includes independent variables regarding socio-demographic characteristics, working environments, relationships with clients, social support network, economic burden, subjective perception of discrimination, and subjective perception of their work.

We first examined the results of Model 1. Socio-demographic characteristics such as gender, age, and household registration were not significantly associated with respondents’ depression levels. However, marital status and education level showed a significant association with depression level. Unmarried home care workers were more likely than married workers to have a higher level of depression, while there were no significant differences among married, divorced, and widowed home care workers. Illiterate home care workers were more likely than other educated home care workers to have a higher level of depression. Respondents who had the highest education level had the lowest level of depression. The subjective perception of health also showed a significant association with level of depression. Respondents who reported being healthier were more likely to report a lower level of depression than their counterparts.

All the independent variables regarding working environment, including annual income, number of rest days per month, type of care work (maternal, child, housekeeper, and cleaner), working hour arrangement (live-in, day-shift, and hourly), living conditions (whether they lived with clients or not),condition of surveillance (whether they were supervised by the clients through camera), and training on emotional regulation strategies (whether they received training on emotional regulation strategies provided by their company), were not significantly associated with the level of respondents’ depression. Moreover, the amount of contact engaged in with friends and family members had no significant association.

However, independent variables regarding their relationships with clients, economic burden, subjective perception of discrimination, and subjective perception of work were found to be significantly associated with the workers’ level of depression. Home care workers who reported having a very good relationship with their clients were more likely to have a lower level of depression than those who reported not having a very good relationship with their clients. Furthermore, home care workers who reported having economic burden were more likely to have a higher level of depression than those who reported not having economic burden. In terms of the subjective perception of discrimination, those who perceived discrimination were more likely to have a higher level of job satisfaction than those who did not perceive discrimination. Regarding the subjective perception of care workers, home care workers who reported liking their care work were more likely to have a lower level of depression than those who reported not liking their work.

## 4. Discussion

This study examined factors that influence the depression level of informal home care workers who are rural migrant women, including socio-demographic characteristics, working environment, relationships with clients, and subjective perception of their care work. These workers provide home-based maternal, child, elderly care, housekeeping, and cleaning services in Shanghai. More than half of these workers are engaged on an hourly schedule, but some still work as live-in workers. Coinciding with the findings of previous studies, most home care workers in Shanghai lack labor contracts [10,11]. Most of them are rural married migrant women who work in the informal sector and have a low level of education. Consistent with the results of previous studies on homecare workers’ social relations, their social relationships with their clients are associated with their depression level [27,28]. Furthermore, workers’ subjective perception of their care work plays a more important role in their mental health. This result is consistent with the previous finding that home care workers might have a positive experience of their work because they find social meaning in their care work [14]. However, working environment is not associated with workers’ depression levels, which is different from the previous results found regarding caregivers in other countries [25,26,27,28].

Moreover, our research contributes to the current discussion on caregivers’ mental health by arguing the following two points. The first is that home care workers’ subjective perspective of their work might be separate from their objective work environment. The other is that home care workers’ subjective perspective of their work might have a greater effect than their objective work environment on their level of depression. These two points have seldom been discussed in previous studies. Filling in this gap, the results of our model show that rural migrant women who have a more positive perspective of their care work might have lower levels of depression.

One’s subjective perception of work might be separated from one’s objective work environment. One reason is that home care workers have their own approaches to subjectivity and agency. They develop their own strategies to deal with the difficulties present in their work and home life. By reconstructing their explanation of their work and home life, they are able to see the positive meaning of their experiences with both aspects of their life [14]. Furthermore, the home care industry needs workers’ emotional devotion to meet the needs of those receiving care. Whether home care workers like engaging in care work influences their emotional condition. The previous literature has shown that having positive feelings toward one’s work can help one avoid emotional exhaustion [33,34,35,36]. Thus, home care workers who like their care work might have more positive feelings than their counterparts. This means that they might be more enthusiastic and fuller of emotional energy, which might reduce their depression level. In contrast, subjective discrimination might damage their confidence and self-esteem, thereby strengthening their emotional burden; this might harm their mental health.

Relationships with clients were found to be significantly associated with home care workers’ depression levels. The results of our model showed that home care workers who have a very bad relationship with their clients have a higher level of depression than those who have a very good relationships with their clients. These findings are consistent with the results of qualitative studies on rural migrant women who provide home care, which conclude that abuse and violence that stem from clients might cause trauma for home care workers [25,26,27,28]. Therefore, relationships with clients still play a significant role in home care workers’ mental health. Some socio-demographic characteristics, such as marriage and education, also show a significant association with depression, while other socio-demographic characteristics, such as gender, age, and household registration, do not show a significant association. The model results demonstrated that illiterate workers’ depression levels are higher than those of workers who are educated. These results are consistent with the findings of the previous literature that low-educated rural migrant women tend to lack the resources to deal with abuse and violence within the workplace, which leads to their anxiety and distress [10]. Furthermore, unmarried home care workers are likely to have a higher level of depression than those who are married. The reason for this outcome is that family members might provide social support when they face discrimination and abuse in their workplace and help them to attenuate their anxiety. In addition, self-reported health had a significant association with home care workers’ depression levels. These results also coincide with the findings of previous studies on the relationship between physical condition and mental health, suggesting that a poor physical condition might result in an emotional burden [42,43].

However, the results of this research showed that the working environment, including income, days of rest per month, living place, type of care provided, working hour arrangement, contractual status, and surveillance, of home care workers has no significant effect on their level of depression in Shanghai, which is not consistent with the findings of previous studies on caregivers conducted in other countries [25,26,27,28]. The reason might be that a positive subjective perception of the working environment helps home care workers to develop the resilience to deal with the difficulties present in their work and life [44,45]. Their negative effect of objective working environment might be relieved by the impact of home care workers’ positive subjective perception of work. The results implied that the objective working environment might have a smaller effect than the home care workers’ subjective perception of work on their level of depression.

In addition, social support networks are not significantly associated with home care workers’ depression levels. This is different from the results of previous studies, which showed that migrant workers tend to rely on social relations with their family members, friends, and folks, who provide them support, to attenuate the anxiety and distress that result from their workplace experiences [23,24]. In contrast, our model showed that the number of monthly contacts is not significantly associated with the depression level of home care workers. The reason for this might be that most home care workers spend a large amount of time with their clients and therefore lack deep communication with their family members, friends, and folks.

## 5. Limitations

This research has some limitations. First, due to utilizing the respondent-driven sampling approach, this research is constrained by the limitations inherent to this method. Although this is the most suitable sampling method for rural migrant women, for whom there is no sampling frame, it is difficult to avoid having some sampling error. Second, the results are difficult to generalize to the national scale because the sample is limited to large cities such as Shanghai. Therefore, the data should be supplemented by conducting similar studies in other small cities. Given that home care workers in other cities might have different work and life experiences, the relationship between their subjective perception of work and their mental health should be deeply explored.

## 6. Conclusions

Examining the depression level of rural migrant women who work as home care workers in Shanghai is important for two reasons. First, the findings are significant to improving the quality of home care provided by rural migrant workers in developing countries, which might influence clients’ satisfaction levels. Second, such a study is essential to promoting home care workers’ mental health, which is closely associated with their work performance, retention, and work rights. Our study used the simplified 10-item Center for Epidemiologic Studies Depression Scale (CES-D 10) to measure home care workers’ level of depression. We analyzed the relationship between home care workers’ depression level and their socio-demographic characteristics, self-reported health, working environments, relationships with clients, social supportive network, economic burden subjective perception of discrimination, and subjective perception of their care work. We found that for home care workers who are rural migrant women, one of the most significant factors that result in a low level of depression is their positive subjective perception of their care work. Our results contribute to the current literature on home care workers in regard to two aspects. The first aspect is that promoting an objective working environment through such factors as income, contract, rest, and living space may not be enough to improve home care workers’ mental health. The second aspect is improving home care workers’ subjective perception of their work. Eliminating the discrimination of their work and giving them more recognition might contribute to them having a more positive perception of their care work, which would in turn strengthen their work identity and relieve their emotional burden and exhaustion. Both of these dimensions of results are relevant to policy-makers such as the All-China Women’s Federation (ACWF) in China and local governments. Due to the future need for home care in China, a more comprehensive understanding of the factors affecting home care workers’ mental health would be helpful for formulating supportive policies that aim to both protect these rural migrant women and guarantee the quality of home care.

## Figures and Tables

**Table 1:** Socio-demographic characteristics, work environment, social relations, economic burden, subjective perception of discrimination, and work.

Variable	
**Gender**, *n* (%)	
Women	972 (97.2)
Men	28 (2.8)
**Age**, *Mean Value* (*SD*)	50 (6.4)
**Household registration**, *n* (%)	
Rural	781 (78.3)
Urban	216 (21.7)
**Marital status**, *n* (%)	
Married	900 (90.0)
Divorced	55 (5.5)
Widowed	39 (3.9)
Unmarried	6 (0.6)
**Level of education**, *n* (%)	
No education	82 (82.0)
Primary school and under	272 (27.2)
Junior high school	467 (46.7)
High school	27 (27)
College and above	25 (25)
**Self-reported health**, *Mean Value* (*SD*)	4.4 (0.7)
**Log (annual income)**, *Mean Value* (*SD*)	4.7 (0.3)
**Number of rest days per month**, *Mean Value* (*SD*)	3.4 (3.1)
**Contractual status**, *n* (%)	
No contract	440 (44.0)
**Type of care**, *n* (%)	
Maternal care	73 (7.3)
Child care	133 (13.3)
Elderly care	285 (28.5)
Housekeeping	361 (36.1)
Cleaning	142 (14.2)
Others	6 (0.6)
**Working hour arrangement**, *n* (%)	
Lived in	342 (34.2)
Day shift	149 (14.9)
Hourly	509 (50.9)
**Living with clients**, *n* (%)	347 (34.7)
**Under surveillance by camera**, *n* (%)	411 (41.4%)
Training on emotional regulation strategies, *n* (%)	356 (35.6)
**Relation with clients**, *n* (%)	
Bad	7 (0.7)
Neutral	99 (9.9)
Good	894 (89.4)
**Numbers of contacts per month**, *Mean Value* (*SD*)	13 (15.3)
Economic burden, *n* (%)	169 (16.9)
**Subjective perception of discrimination**, *n* (%)	489 (48.9%)
**Subjective perception of care work**, *n* (%)	
Not like care work	89 (8.9)
neutral	193 (19.3)
Like care work	718 (71.8)

**Table 2:** Average score of respondents’ depression in each item of CES-D10.

Items of CES-D10	Average Score (SE)
I was bothered by things that usually do not bother me	0.491 (0.754)
I have trouble keeping my mind on what I was doing	0.315 (0.645)
I feel depressed	0.489 (0.784)
I felt that everything I did was an effort	0.385 (0.737)
I felt hopeful about the future (reverse scored)	0.719 (1.002)
I felt fearful	0.157 (0.486)
My sleep was restless	0.726 (1.012)
I was happy (reverse scored)	0.673 (0.875)
I felt lonely	0.403 (0.796)
I could not “get going”	0.292 (0.661)

**Table 3:** Multiple linear regression analysis of home care workers’ depression.

	Model 1		
Independent Variables	Coefficient	SE	*p*
**Gender** (ref. women)			
men	−0.022	0.887	0.469
**Age** **Household Registration** (ref. rural)	0.016 0.026	0.026 0.375	0.645 0.412
**Level of Education** (ref. no education)			
Primary school	−0.140	0.562	0.008
Junior high school	−0.143	0.558	0.015
High school	−0.143	0.677	0.006
College and above	−0.071	1.152	0.046
**Marital status** (ref. married)			
Divorced	0.011	0.618	0.726
Widowed	0.012	0.745	0.697
Unmarried	0.066	2.126	0.030
**Self-reported health**	−0.175	0.184	<0.001
**log (annual income)**	−0.002	0.527	0.939
**Number of rest days per week**	−0.060	0.054	0.086
**Contractual status** (ref. no contract)	0.019	0.313	0.555
**Type of care** (ref. maternal care)			
Child care Elderly care Housekeeping Cleaning	−0.026 0.019 −0.007 0.041	0.679 0.722 0.698 0.807	0.587 0.780 0.923 0.499
**Type of work** (ref. live-in)			
Day shift Hourly	0.010 0.016	1.027 1.003	0.899 0.878
**Living with clients** (ref. not live-in)	0.126	0.967	0.194
**Surveillance** (ref. no surveillance)			
Surveillance by camera	0.002	0.309	0.958
Unclear	−0.032	0.539	0.778
**Training on emotional regulation strategies**	0.009	0.329	0.778
**Relation with clients** (ref. bad)			
Neutral Good	−0.392 −0.439	1.786 1.736	<0.001 <0.001
**Numbers of contacts per month**	−0.022	0.009	0.467
**Economic burden**	0.152	0.401	<0.001
**Subjective perception of discrimination**			
(ref. no subjective perception of discrimination)	0.228	0.296	<0.001
**Subjective perception of care work** (ref. not like care work)			
Neutral Like care work	−0.201 −0.326	0.579 0.517	<0.001 <0.001
**adj.R^2^**	0.265		

## Data Availability

Data are not available due to confidentiality.
